# Prostate cancer-derived CCN3 induces M2 macrophage infiltration and contributes to angiogenesis in prostate cancer microenvironment

**DOI:** 10.18632/oncotarget.1570

**Published:** 2014-02-04

**Authors:** Po-Chun Chen, Hsu-Chen Cheng, John Wang, Shin-Wei Wang, Huai-Ching Tai, Chiao-Wen Lin, Chih-Hsin Tang

**Affiliations:** ^1^ Graduate Institute of Basic Medical Science, China Medical University, Taichung, Taiwan; ^2^ Department of Medical Research, Chung Shan Medical University Hospital, Chung Shan Medical University, Taichung, Taiwan; ^3^ Department of Life Sciences, National Chung Hsing University, Taichung, Taiwan; ^4^ Department of Pathology, Taichung Veterans General Hospital, Taichung, Taiwan; ^5^ Department of Medicine, Mackay Medical College, New Taipei City, Taiwan; ^6^ Department of Urology, National Taiwan University Hospital, Taipei, Taiwan; ^7^ Graduate Institute of Clinical Medicine, National Taiwan University, Taipei, Taiwan; ^8^ Institute of Oral Sciences, Chung Shan Medical University, Taichung, Taiwan; ^9^ Department of Dentistry, Chung Shan Medical University Hospital, Taichung, Taiwan; ^10^ Department of Pharmacology, School of Medicine, China Medical University, Taichung, Taiwan; ^11^ Department of Biotechnology, College of Health Science, Asia University, Taichung, Taiwan

**Keywords:** CCN3, VEGF, Prostate cancer, M2 macrophage, angiogenesis

## Abstract

Tumor-associated macrophages (TAMs) are M2-polarized macrophages that infiltrate the tumor microenvironment and promote tumorigenesis. However, the mechanisms by which TAMs modulate prostate cancer (PCa) growth are poorly understood. Here, we found that expression of Nephroblastoma Overexpressed (NOV/CCN3) is upregulated in PCa cells and correlated with M2 macrophage infiltration. RAW264.7 macrophage migration was induced by conditioned media (CM) from various PCa cells in proportion to the cellular level of CCN3 expression and was inhibited by an anti-CCN3 neutralizing antibody. CCN3 and PCaCM treatment skewed RAW264.7 cell differentiation from an M1 phenotype to an M2 phenotype. PCa-derived CCN3 induced focal adhesion kinase (FAK)/Akt/NF-κB signaling in RAW264.7 cells, which resulted in VEGF expression and subsequently increased tube formation in endothelial progenitor cells. Finally, PCa-secreted CCN3 stimulated RAW264.7 cells and promoted angiogenesis in the chick chorioallantoic membrane assay (CAM), and increased tumor growth and tumor-associated angiogenesis in a PCa xenograft mouse model. Our results indicate that PCa-secreted CCN3 can recruit macrophages and skew their differentiation to an M2 phenotype. In turn, CCN3-stimulated macrophages contribute to VEGF-dependent angiogenesis. This study reveals a novel mechanism by which TAMs enhance PCa angiogenesis and identifies a potential therapeutic target for PCa.

## INTRODUCTION

Prostate cancer (PCa) is the most commonly diagnosed malignancy in the United States and other Western countries [1, 2]. Surgery is the usual therapeutic method at the early stages of PCa, but the high rate of secondary metastasis makes PCa the second leading causes of cancer-related mortality.

The tumor microenvironment is a complex system composed of many cell types, including endothelial cells, smooth muscle cells, fibroblasts, and inflammatory cells such as macrophages and dendritic cells [3]. The interaction between tumor cells and cells in their microenvironment is known to be crucial for malignant progression [3]. Macrophages are the most abundant cells resident in the tumor microenvironment, and one subpopulation is recruited to the tumor microenvironment by tumor-secreted cytokines, where they influence tumor progression [4]. The phenotype of these tumor-associated macrophages (TAMs) can influence their effects on tumor growth: M1 macrophages generally are anti-tumoral, whereas M2 macrophages exert pro-tumoral effects [5]. Within the tumor microenvironment, TAM-induced angiogenesis has been associated with cancer progression and proliferation [6, 7]. This is supported by recent demonstrations that TAMs produce angiogenic factors such as vascular endothelial growth factor (VEGF) and platelet-derived endothelial growth factor [7, 8].

Nephroblastoma Overexpressed (NOV/CCN3) was first discovered in chicken myeloblastosis-associated virus-induced nephroblastomas [9]. NOV is a member of the CCN gene family, which includes the cysteine-rich 61 (CYR61) and connective tissue growth factor (CTGF) genes. A previous study showed that CCN3 expression is upregulated in PCa cells and human PCa patients [10]. In addition, CCN3 is overexpressed in malignant PCa cell lines and increases their ability to migrate and metastasize to the bone [11]. Furthermore, our previous study showed that CCN3 modulates the microenvironment of bone-metastasized PCa by promoting osteoclastogenesis [12]. These data indicate that CCN3 plays an important role in PCa progression.

CCN3 is a multifunctional cytokine that regulates signals from the extracellular matrix (ECM), and expression of CCN3 correlates with tumorigenesis [11, 12]. Recent study shows that CCN3 is a novel angiogenic regulator that promotes angiogenesis in vivo [13]. Nevertheless, it is unknown whether CCN3 increases TAM infiltration or induces angiogenesis in PCa. Here, we provide the first evidence that PCa-derived CCN3 recruits macrophages and contributes to tumor-associated angiogenesis in human PCa. In addition, we show that M2 macrophages are abundant in the tumor margins of PCa specimens. PCa-derived CCN3 skewed macrophage differentiation toward the M2 phenotype, and subsequently promoted macrophage production of the angiogenic factor VEGF. CCN3-induced VEGF production and angiogenesis were mediated by focal adhesion kinase (FAK)/Akt/NF-κB signaling in the M2 macrophages. Finally, we show that PCa-secreted CCN3 stimulated RAW264.7 cells and subsequently increased angiogenesis in vivo. In conclusion, these results indicate that PCa-derived CCN3 regulates TAM function to promote angiogenesis in the tumor environment.

## RESULTS

### PCa-secreted CCN3 increases CD206-positive M2 macrophage infiltration into the tumor microenvironment

**Fig 1:**
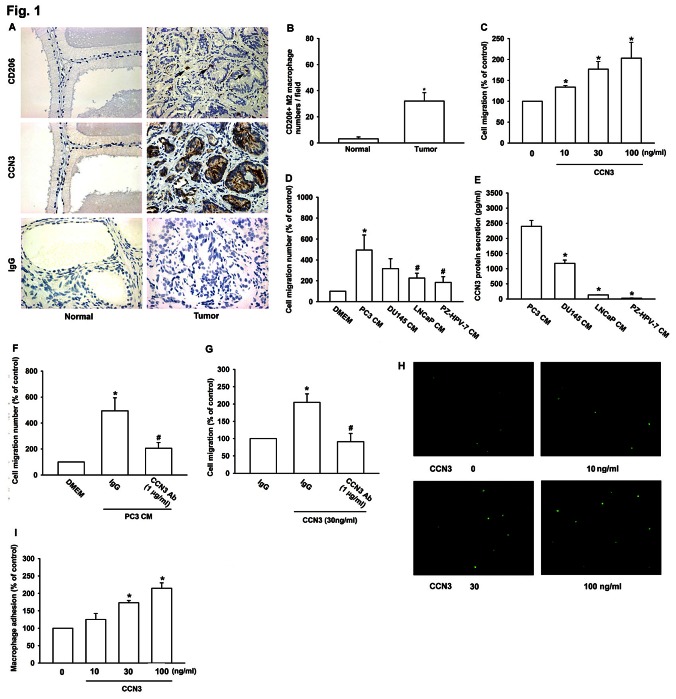
M2 Macrophage infiltration in human PCa specimens is related to CCN3 expression (A) Representative IHC images of CD206+ macrophages and CCN3 expression in paraffin-embedded sections of normal prostate tissue and human PCa specimens. Macrophage infiltration was observed as CD206 staining in the tumor region (right). IgG staining was used as a negative control. (B) Quantification of CD206+ staining by IHC. The number of CD206+ macrophages was counted in 5 independent fields per section. (C) RAW264.7 cells were incubated with CCN3 (0, 10, 30, or 100 ng/ml) in Transwells and migration was measured 24 h later. (D) Conditioned media (CM) from PC3, DU145, LNCaP, and PzHPV-7 cells were placed in the lower chambers of Transwells and migration of RAW264.7 cells was measured 24 h later. (E) Total protein secreted in CM from PC3, DU145, LNCaP, and PzHPV-7 cells were prepared, and CCN3 expression level was examined by ELISA. (F) CM from PC3 cells was placed in the lower chamber of Transwells in the presence of a neutralizing antibody against CCN3 (1 μg/ml) or an isotype control antibody (IgG). Migration of RAW264.7 was measured 24 h later. (G) RAW264.7 cells were treated as (fig. 1C) with neutralizing antibody against CCN3 (1 μg/ml) or an isotype control antibody (IgG) in Transwells and migration was measured 24 h later. (H) Representative micrographs of adhesion between PC3 and RAW264.7 cells after preincubation of PC3 cells with CCN3 (0, 10, 30, or 100 ng/ml) for 24 h. (I) Quantification of the adherent RAW264.7 cells per well in (H). Results are expressed as the mean ± SEM of triplicate samples. In (B), *P < 0.05 compared to the normal group. In (C, G, I), *P < 0.05 compared to the control group. In (D, F), *P < 0.05 compared to treatment with the control medium (DMEM) and #P < 0.05 compared to treatment with PC3-CM. In (E), *P < 0.05 compared to PC3 CM.

Previous study showed that TAMs display an M2 phenotype and promote tumor progression [14]. To determine if tumor-infiltrated macrophages were M2 polarized in human PCa tumors, we analyzed CD206 (a M2 macrophage marker) expression in 21 human PCa specimens and adjacent normal tissues using immunohistochemistry (IHC). We found that numerous CD206^+^ macrophages had infiltrated to the tumor stroma, but not the adjacent normal tissues (fig. 1a and 1b). We previously reported that CCN3 expression by human PCa cells was positively correlated with malignancy and increased metastasis [11], and which might contribute to macrophage infiltration in PCa. Consistent with this, IHC of CCN3 showed strong staining in tumor areas with high macrophage infiltration (fig. 1a), suggesting that M2 macrophages may be recruited to the tumor microenvironment by PCa-derived CCN3. To test this, we performed Transwell migration assays with purified CCN3 and the macrophage cell line RAW264.7. The results showed that treatment with CCN3 (0–100 ng/ml) obviously increase RAW264.7 macrophage migration in a dose-dependent manner (fig. 1c). We also examined the RAW264.7 migration response to conditioned media (CM) from 4 PCa cell lines; PC3, DU145, LNCaP, and PzHPV-7. We found that all of the PCa-derived CM induced RAW264.7 migration, and the degree of migration correlated with the expression level of CCN3 by the individual PCa and normal prostate epithelial cell lines, as described previously [10, 11] (fig. 1d and 1e) Furthermore, macrophage migration induced by PC3-CM and recombinant CCN3 was reduced by pretreatment with a CCN3-neutralizing antibody (Cat.#: S1262, Epitomics) (fig. 1f and fig. 1g). Previous studies have shown that adhesion to tumor cells can activate macrophages [15, 16]. Thus, we hypothesized that tumor cells might not only recruit macrophages but also retain them in the tumor microenvironment. In support of this, we found that macrophage–PCa adhesion was markedly increased by incubation of PCa cells with CCN3 (0–100 ng/ml; fig. 1h and 1i). In summary, these results showed that tumor-secreted CCN3 increased macrophage migration and adhesion to PCa cells.

### PCa-derived CCN3 polarizes innate RAW264.7 macrophages to the M2 phenotype

**Fig 2:**
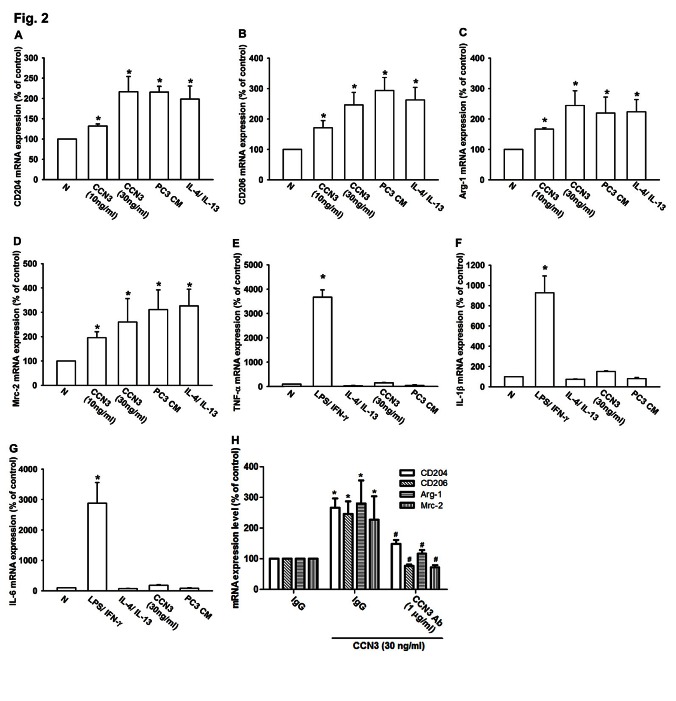
CCN3 promotes innate RAW264.7 macrophages toward M2 phenotype polarization (A-D) CCN3 induces M2 macrophage marker expression in innate RAW264.7 macrophages. RAW264.7 macrophages were incubated for 24 h with CCN3 (0, 10 or 30 ng/ml), PC3-CM, or IL-4 (20 ng/ml) plus IL-13 (20 ng/ml) as a positive control. Total RNA was extracted and CD204, CD206, Arg-1, and Mrc-2 mRNA expression levels were examined by qPCR. (E-G) CCN3 does not increase the expression of M1 macrophage proinflammatory cytokines in RAW264.7 macrophages. RAW264.7 macrophages were incubated for 24 h with CCN3 (30 ng/ml), PC3-CM, LPS (50 ng/ml) plus IFN-γ (20 ng/ml), or with IL-4 (20 ng/ml) plus IL-13 (20 ng/ml). Total RNA was extracted and TNF-α, IL-1β, and IL-6 mRNA expression levels were examined by qPCR. (H) RAW264.7 were incubated with CCN3 (30 ng/ml) in the presence of neutralizing antibody against CCN3 (1 μg/ml) or an isotype control antibody (IgG) for 24 hr. Total RNA was extracted and CD204, CD206, Arg-1, and Mrc-2 mRNA expression levels were examined by qPCR. Results are expressed as the mean ± SEM of triplicate samples. *P < 0.05 compared with the control treatments. #P < 0.05 compared to treatment with CCN3 treatment gruup.

TAMs are often characterized as displaying an M2 phenotype [14, 17], which can be determined using several previously identified M2 macrophage markers, including CD204, CD206, mannose receptor C-2 (Mrc-2), and arginase-1 (Arg-1) [18-20]. RAW264.7 macrophages have been described as “innate” macrophages that can differentiate into M1 or M2 macrophages [21]. To determine whether PCa-secreted CCN3 can promote innate RAW264.7 differentiation into M2 macrophages, we examined the expression of M2 macrophage markers. Treatment of innate RAW264.7 cells with CCN3 (0–30 ng/ml), PC3-CM, or IL-4 plus IL-13 (positive control) markedly increased the expression of M2 macrophage markers (CD204, CD206, Arg-1, and Mrc-2; fig. 2a–d). Expression of a particular pattern of inflammatory cytokines is an important characteristic of polarized macrophages. M1 macrophages are known to secrete proinflammatory cytokines such as TNF-α, IL-1β, and IL-6 [22]. Results showed that the positive control treatment of LPS plus IFN-γ increased the expression of M1-type inflammatory cytokines (TNF-α, IL-1β and IL-6), but this was not observed with either CCN3 (30 ng/ml) or PC3-CM (fig. 2e–g). Moreover, pretreatment with CCN3 antibody abolished CCN3-promoted M2 markers expression in RAW264.7 (fig. 2h). We also found that treatment with LPS plus IFN-γ attenuated the expression of M2 macrophage markers in innate RAW264.7 macrophages, but pretreatment of cells with CCN3 reversed the LPS/IFN-γ effect (fig. S1a–d). Consistent with this, pretreatment with CCN3 markedly decreased the LPS/IFN-γ–induced expression of M1 macrophage markers (fig. S1e–g). Taken together, these results showed that PCa-secreted CCN3 could polarize innate RAW264.7 cells to an M2 macrophage phenotype. This shift of phenotype could alter proinflammatory cytokine expression by the macrophages, which could suppress the immune response within the tumor microenvironment.

### PCa-derived CCN3 increases VEGF expression and regulates angiogenesis in RAW264.7 macrophage

**Fig 3:**
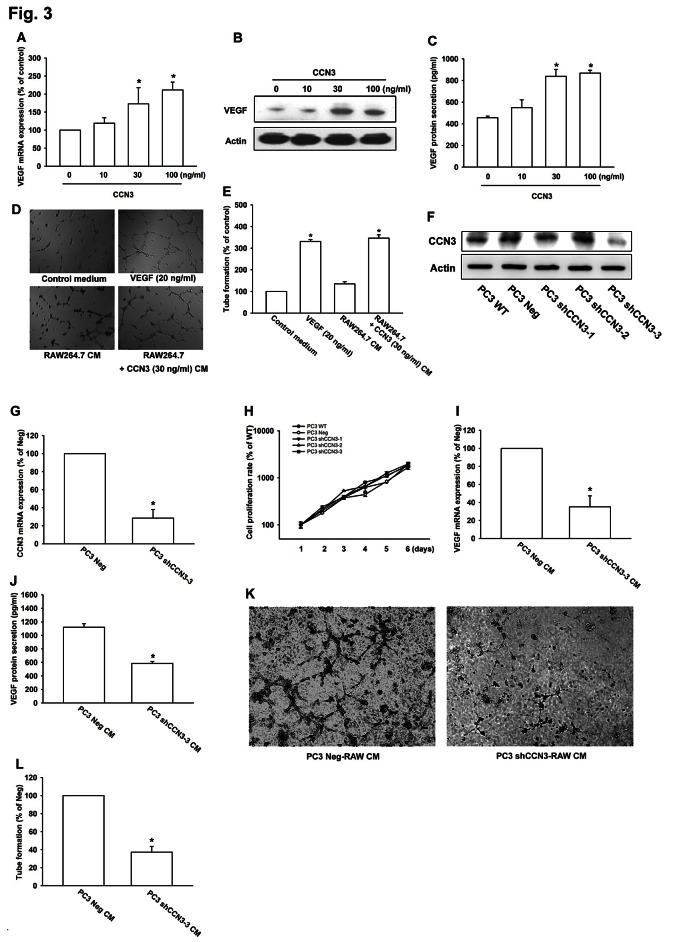
CCN3 increases VEGF expression and regulates angiogenesis (A) RAW264.7 macrophages were incubated with CCN3 (0, 10, 30, or 100 ng/ml) for 24 h and VEGF mRNA levels were measured by qPCR. (B) RAW264.7 macrophages were incubated with CCN3 (0, 10, 30, or 100 ng/ml) for 24 h and cell lysates were prepared. The level of VEGF in cell lysates was determined by western blot analysis. Anti-actin was used as a loading control. (C) RAW264.7 macrophages were treated as described in (B) and the CM were collected. VEGF secretion was quantified by ELISA. (D) RAW264.7 macrophages were treated with or without CCN3 (30 ng/ml) for 24 h and the CM were collected. EPC cells were seeded onto Matrigel and incubated with the RAW264.7 CM for 6 h. The capillary-like structures formed in EPCs were photographed. VEGF (20 ng/ml) was used as a positive control. (E) Quantification of tube-like structure per field in (D). (F, G) CCN3 protein and mRNA expression in PC3 cells expressing control (Neg) or CCN3-targeted shRNAs were examined by western blotting or qPCR. (H) PC-3 shCCN3 stable clone cells which stably expressing shRNA constructs were seeded as monolayers and counted daily. Cells (10^4^) were reseeded after each count, and the cell numbers were plotted. (I) RAW264.7 macrophages were incubated with CMs collected from PC3 Neg and PC3 shCCN3-3 cells for 24 h and VEGF mRNA levels were measured by qPCR. (J) RAW264.7 macrophages were treated as described in (I) and the RAW264.7 CM were collected. VEGF secretion was quantified by ELISA. (K) RAW264.7 macrophages were incubated with PC3 Neg CM or PC3 shCCN3-3 CM for 24 h and the RAW264.7 CM were collected. EPC cells were seeded onto Matrigel and incubated with these two CM for 6 h. The capillary-like structures formed in EPCs were photographed. (L) Quantification of tube-like structure per field in (K). Results are expressed as the mean ± SEM of triplicate samples. In (A, C, E), *P < 0.05 compared with the control. In (G, I, J, L), *P < 0.05 compared to treatment with the PC3 Neg group.

Previous studies have indicated that TAMs regulate angiogenesis in the tumor environment by production of the angiogenic factor VEGF [7, 8, 23]. Our experiments showed that treatment of RAW264.7 macrophage with CCN3 (0–100 ng/ml) increased VEGF expression and secretion in a dose-dependent manner (fig. 3a–c). An earlier study showed that endothelial cells must undergo migration, proliferation, and tube formation as part of the angiogenic process [24]. Next, we used an in vitro endothelial progenitor cell (EPC) model to investigate whether CCN3 could induce VEGF-dependent angiogenesis in macrophage. For this, EPC cells were cultured with control medium, CM from RAW264.7 cells treated with or without CCN3 (30 ng/ml), or VEGF (positive control). The CM from CCN3-treated, but not untreated RAW264.7 cells induced tube formation by the EPCs (fig. 3d and 3e). Finally, to clarify the role of CCN3 in VEGF-dependent angiogenesis in macrophage, we generated PC3 cells stably expressing CCN3 shRNAs (PC3 shCCN3-1, shCCN3-2 and shCCN3-3) or the empty vector (PC3 Neg), and found that CCN3 protein and mRNA expression were effectively inhibited in the PC3 shCCN3-3 stable clone (fig. 3f and 3g). However, the result showed that CCN3 expression did not affect cell viability (fig. 3h). Finally, we incubated RAW264.7 macrophage with the CMs collected from these two PC3 stable cell lines (PC3 Neg and PC3 shCCN3-3) for 24 h. After incubating, the VEGF expression and angiogenesis were accessed. Knockdown of CCN3 in PC3 cells was dramatically to reverse the effect of PC3-CM on the induction of VEGF expression and promoting angiogenesis in RAW264.7 macrophages (fig. 3i-3l). These results suggested that CCN3 could regulate angiogenesis by inducing VEGF production in TAMs within the tumor microenvironment.

### The FAK/Akt/NF-κB signaling pathway is involved in CCN3-induced VEGF expression and promotion of angiogenesis in RAW264.7 macrophages

**Fig 4:**
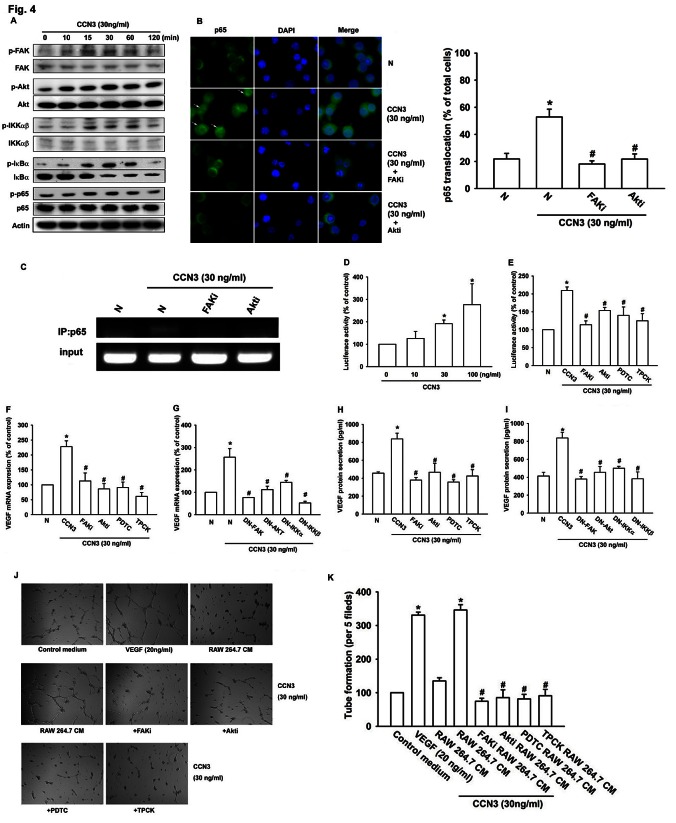
CCN3 increases VEGF expression and angiogenesis by stimulating FAK/Akt/NF-κB signaling in RAW264.7 macrophages (A) RAW264.7 macrophages were incubated with CCN3 (30 ng/ml) for the indicated times, and phosphorylation of FAK, Akt, IKKα/β, IκBα, and p65 was determined by western blot analysis. (B) RAW264.7 macrophages were pretreated for 30 min with FAKi (10 μM), Akti (1 μM), or vehicle (N) and then stimulated with CCN3 (30 ng/ml) for 1 h. Cells were stained with anti-p65 antibody and analyzed by fluorescence microscopy. White arrows show p65 nuclear translocation. Nuclei were counterstained with DAPI. Representative confocal microscopy images were shown. The quantification of p65 nuclear translocation was shown in right panel (% of total cell numbers per 5 fields). (C) RAW264.7 macrophages were treated as described in (B) and chromatin immunoprecipitation assays were performed using anti-p65 antibody. One percentage of the precipitated chromatin was analyzed to verify equal loading (input). (D) RAW264.7 macrophages were transfected for 24 h with an NF-κB promoter reporter plasmid, incubated with CCN3 (0, 10, 30, or 100 ng/ml) for a further 24 h, and then analyzed for luciferase activity. (E) RAW264.7 macrophages were transfected for 24 h with an NF-κB promoter reporter plasmid, pretreated with FAKi (10 μM), Akti (1 μM), PDTC (10 μM), or TPCK (1 μM) for 30 min, incubated with CCN3 (30 ng/ml) for a further 24 h, and then analyzed for luciferase activity. (F) RAW264.7 macrophages were pretreated with FAKi (10 μM), Akti (1 μM), PDTC (10 μM), or TPCK (1 μM) for 30 min and then incubated with CCN3 (30 ng/ml) for 24 h. Total RNA was extracted and VEGF mRNA levels were examined by qPCR. (G) RAW264.7 macrophages were transfected with dominant-negative (DN) mutant FAK, Akt, IKKα, or IKKβ for 24 h and then stimulated with CCN3 (30 ng/ml) for 24 h. N represents a control transfection. Total RNA was extracted and VEGF mRNA levels were examined by qPCR. (H, I) Raw264.7 macrophages were treated as described in (F, G) and VEGF protein secretion were determined by ELISA. (J, K) RAW264.7 macrophages were pretreated with FAKi (10 μM), Akti (1 μM), PDTC (10 μM), or TPCK (1 μM) for 30 min and then stimulated with CCN3 (30 ng/ml) for 24 h. CM were collected from the cultures and applied to EPCs for 6 h. VEGF (20 ng/ml) was used as a positive control. The capillary-like structures formed in EPCs were photographed and the number per field was counted. Results are expressed as the mean ± SEM of triplicate samples. *P < 0.05 compared with control, #P < 0.05 compared with CCN3 treatment.

The CCN proteins stimulate intracellular signaling pathways through cell surface integrin receptors [25, 26]. We, therefore, analyzed signaling pathways that are known to be regulated by integrin receptors and might regulate VEGF expression in macrophages. Western blot analysis of CCN3-treated RAW264.7 cell extracts showed a time-dependent increase in the phosphorylation of FAK, Akt, IKKα/β, IκBα, and NF-κB subunit p65 signaling proteins (fig. 4a). NF-κB activation was further evaluated by analysis of p65 nuclear translocation, ChIP assay, and NF-κB luciferase reporter assays. Stimulation of RAW264.7 macrophages with CCN3 increased p65 translocation into the nucleus, p65 binding to the NF-κB element on the VEGF promoter, and NF-κB luciferase reporter activity. These effects were greatly reduced by pretreatment of RAW264.7 cells with a FAK inhibitor, an Akt inhibitor, or with either of 2 NF-κB inhibitors, PDTC and TPCK (fig. 4b–4e). Moreover, pretreatment of RAW264.7 macrophages with these inhibitors or transfected with dominant-negative (DN) mutant of FAK, Akt, IKKα, or IKKβ abolished CCN3-stimulated VEGF expression (fig. 4f-4i). Finally, pretreatment with these inhibitors abolished of tube formation by EPCs exposed to CM from CCN3-stimulated RAW264.7 cells (fig. 4j and 4k). These results suggest that CCN3 induces VEGF expression and regulation of angiogenesis by activating FAK/Akt/NF-κB signaling in RAW264.7 macrophages.

### Knockdown of CCN3 in PCa cells inhibits RAW264.7-promoted angiogenesis and tumor growth in vivo

**Fig 5:**
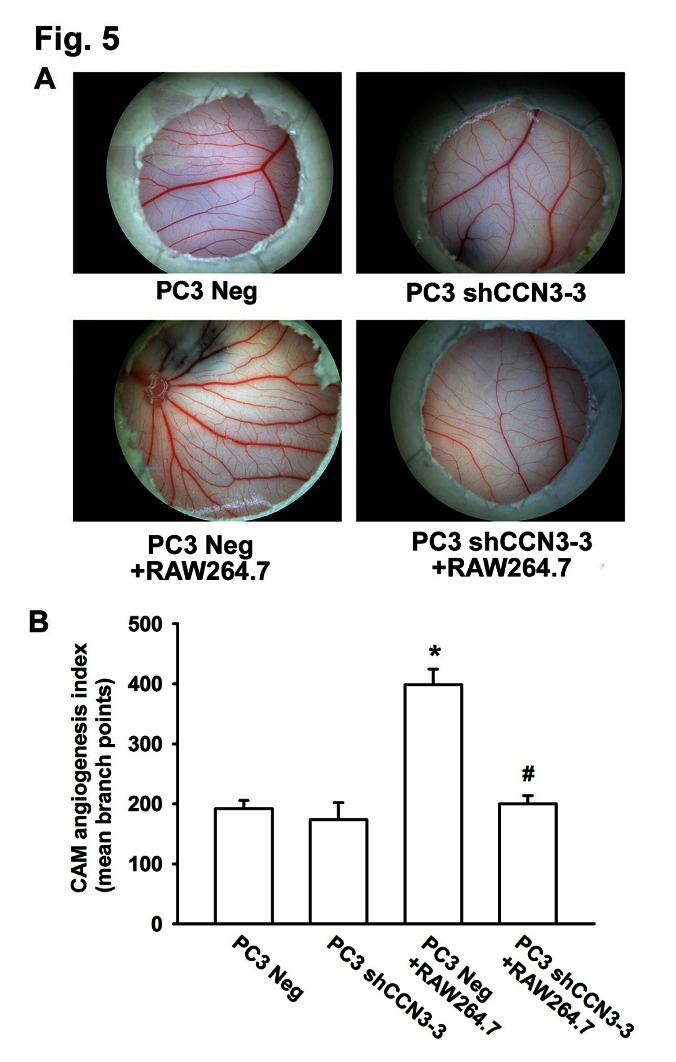
PCa-secreted CCN3 increases RAW264.7 macrophage-stimulated angiogenesis in vivo (A) PC3 Neg, PC3 shCCN3-3, RAW264.7, PC3 Neg plus RAW264.7, or PC3 shCCN3-3 plus RAW264.7 cells were suspended in Matrigel and placed onto chick chorioallantoic membranes (CAMs). On developmental day 12, the CAM arteriosus branches in each treatment group were photographed. (B) Quantification of the branch number in the CAM assay. Results are expressed as the mean ± SEM of triplicate samples. *P < 0.05 compared with the PC3 Neg group. #P < 0.05 compared with PC3 Neg + RAW264.7 group.

**Fig 6:**
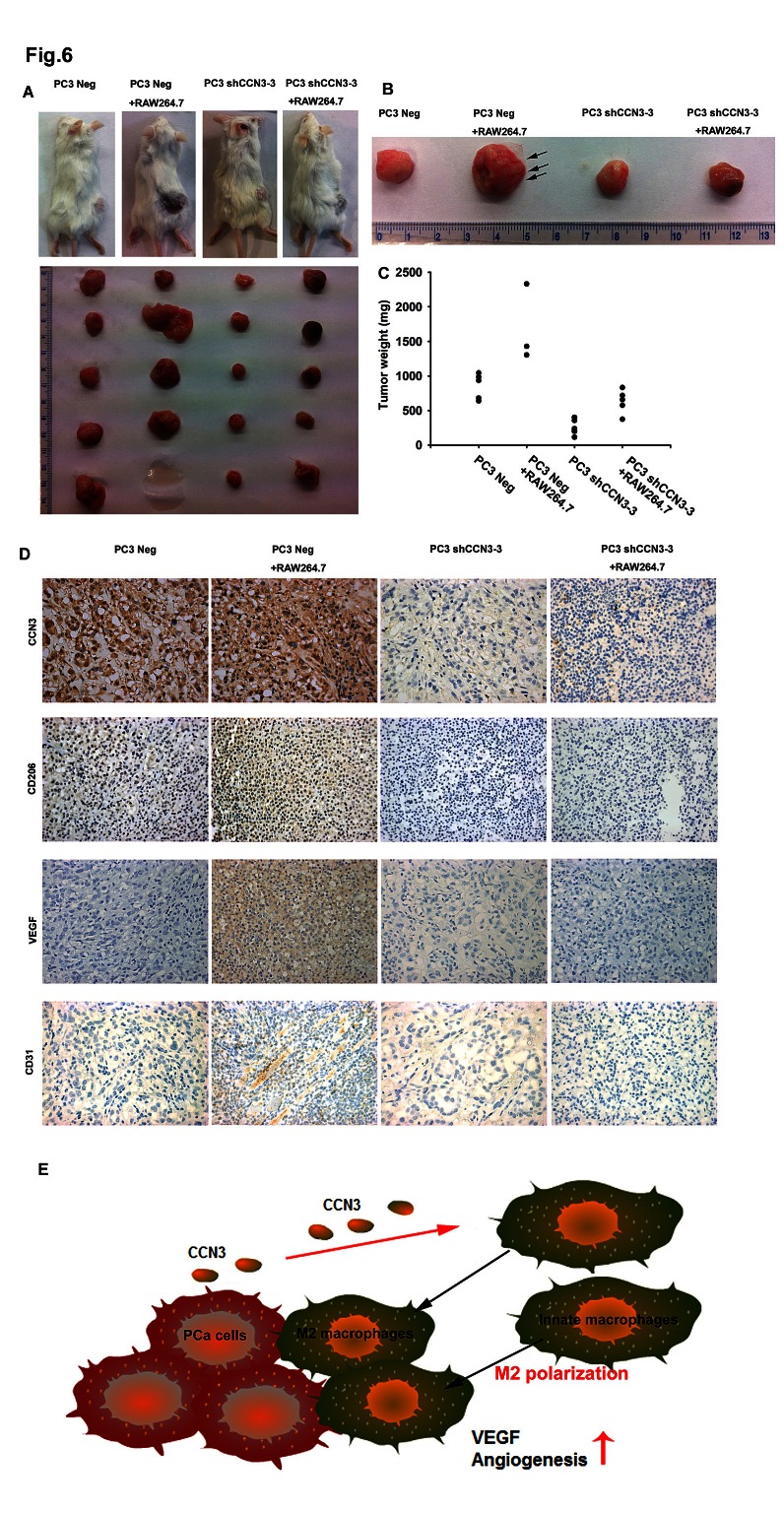
Knockdown of CCN3 in PCa cells inhibits RAW264.7-promoted angiogenesis and tumor growth in a mouse model (A) PC3 Neg and PC3 shCCN3-3 cells, alone or mixed with RAW264.7 macrophages, were suspended in Matrigel and injected subcutaneously into SCID mice. After 28 days, the mice were sacrificed and the tumors excised. (B) Representative capillaries (black arrow) in the tumors. (C) Quantification of tumor weights. (D) Representative IHC images of CCN3, CD206, VEGF and CD31 staining in paraffin-embedded sections of the tumors. (E) Diagrammatic model for the role of CCN3 in prostate cancer tumor microenvironment. PCa-secreted CCN3 induces macrophage infiltration and skew macrophages from an M1 to an M2 phenotype, which is likely to create a more immunosuppressive tumor microenvironment. CCN3 increases VEGF expression and regulates angiogenesis through the FAK/Akt/NF-κB signaling pathway in macrophages. These results all support the conclusion that PCa-secreted CCN3 modulates the tumor microenvironment by regulating TAM infiltration.

Finally, we examined angiogenesis with the chick embryo chorioallantoic membrane (CAM) assay. PC3 Neg or PC3 shCCN3-3 cells were suspended in Matrigel, alone or with RAW264.7 macrophages, and seeded on the CAM surface for 4 days. As expected, the CAMs incubated with PC3 Neg plus RAW264.7 cells showed significantly enhanced angiogenesis compared with CAMs incubated with the PC3 Neg cells alone. In contrast, reduction of angiogenesis was evident in CAMs incubated with PC3 shCCN3-3 cells plus RAW264.7 macrophages (fig. 5). We also investigated whether CCN3 secreted from PCa could stimulate RAW264.7 macrophage infiltration and subsequent VEGF-dependent angiogenesis and tumor growth in a mouse xenograft model. For this, PC3 Neg or PC3 shCCN3-3 cells were suspended in Matrigel with or without RAW264.7 macrophages and injected subcutaneously into SCID mice. We found that mice injected with PC3 Neg plus RAW264.7 cells had larger tumors than mice injected with PC3 Neg cells alone (fig. 6a and 6c). Moreover, knockdown of CCN3 expression in PC3 cells dramatically reduced RAW264.7-mediated tumor growth (fig. 6a and 6c). Our IHC results showed that decreased expression level of CCN3 in PC3 shCCN3-3 xenograft tumors in mice. In addition, the result showed weak CD206+ staining in PC3 Neg cells and moderate staining in PC3 Neg + RAW264.7-injected mice but no signal in PC3 shCCN3-3 cells even plus RAW264.7 cells. These results confirmed our in vitro findings (fig. 6d, upper 2 panels). Furthermore, tumors from the PC3 Neg + RAW264.7-injected mice showed increased blood vessel growth compared with the tumors from mice injected with PC3 Neg, PC3 shCCN3-3, or PC3 shCCN3-3 + RAW264.7 cells, as shown by VEGF and CD31 IHC (fig. 6b and 6d). Collectively, these results show that CCN3, a PCa-secreted factor, increased M2 macrophage polarization, TAM-promoted angiogenesis and tumor growth in the PCa microenvironment in vivo.

## DISCUSSION

CCN3 is a multifunctional cytokine that signals between the cell and the ECM. Recent studies have shown a correlation between CCN3 expression and tumor progression in many cancers [27-29], and CCN3 has been proposed to increase the migration of PCa cells by influencing ICAM-1 expression [30]. Moreover, CCN3 promotes PCa bone metastasis by modulating the tumor–bone microenvironment [12]. Macrophages are highly plastic cells that can infiltrate and be educated within the tumor environment [31], and numerous reports have suggested that tumors should be viewed as complex microenvironments, rather than as individual cancer cells [32]. In this study, we demonstrated that PCa-secreted CCN3, an ECM regulator, could increase macrophage infiltration, M2 macrophage polarization, and VEGF production in the tumor microenvironment. We also found that CCN3 increased VEGF expression by activating the FAK/Akt/NF-κB signaling pathway. Moreover, knockdown of CCN3 in PCa cells inhibited RAW264.7-promoted angiogenesis and tumor growth in vivo. The results of our study, derived from clinical specimens, in vitro cell experiments, and in vivo animal models, indicate that PCa-secreted CCN3 polarizes innate macrophages toward an M2 phenotype and subsequently promotes VEGF-dependent angiogenesis in the PCa microenvironment.

It is generally recognized that the tumor microenvironment is a critical regulator of tumor growth and progression. Recent results indicate that the surrounding stroma and infiltrating inflammatory cells play an important role in tumor progression [33, 34]. Macrophages are the most abundant inflammatory cells in the tumor environment. Although previous studies have shown that macrophages readily infiltrate to PCa tumor margins [35], our study is the first description of TAMs (M2 polarized macrophages) that promote tumor angiogenesis in the PCa tumor microenvironment. Our IHC results showed increased numbers of CD206+ M2 macrophages at the tumor margins in PCa specimens (fig. 1a and 1b), and tumor CCN3 expression correlated with M2 macrophage infiltration (fig. 1a). These data suggest that M2 polarized macrophages may be recruited by tumor-secreted CCN3 and interact with cancer cells in the tumor environment. Previous reports showed that CCN3 serves as a chemoattractant and increases the migration of many cell types [11, 26, 36]. However, this has not previously been demonstrated for macrophages, and we show for the first time that CCN3 stimulates migration of RAW264.7 macrophages (fig. 1c). Moreover, we found that macrophage migration induced by CM from various PCa cell lines correlated with the relative CCN3 expression levels, as previously reported [11]. Notably, the most malignant PCa cell line, PC3, had the highest CCN3 expression level and the greatest effect on macrophage migration (fig. 1d and fig. 1e). As expected, PC3-CM and recombinant CCN3-induced macrophage migration were attenuated by pretreatment with a CCN3-neutralizing antibody (fig. 1f and fig. 1g). We also hypothesized that tumor-infiltrating macrophages might be retained in the tumor microenvironment, which was supported by our results showing that CCN3 increased the adhesion between macrophages and PC3 cells (fig. 1h and 1i). These results demonstrated that tumor-secreted CCN3 stimulated macrophage tumor infiltration and adhesion to PCa.

It is well established that alternatively activated (or “M2 polarized”) macrophages infiltrate the tumor margin and modulate the tumor microenvironment by attenuating the anti-tumor immune response, remodeling the ECM, and enhancing angiogenesis [6, 37]. Our results described a novel secreted factor that could polarize innate macrophage to an M2 phenotype (fig. 2a-2g). Moreover, pretreatment with CCN3 antibody reversed the CCN3-derived M2 polarization (fig. 2h). Additionally, we found that CCN3 could also switch M1 macrophages to an M2 phenotype. The shift from the M1 to the M2 phenotype inhibited proinflammatory cytokine expression, which would be expected to attenuate the immune response (fig. S1). In conclusion, we found that PCa-secreted CCN3 educated an innate macrophage line toward an M2 phenotype and repressed the immune response in vitro.

It is generally agreed that angiogenesis is crucial for tumor growth and the formation of metastases. Numerous studies have shown that TAMs play a critical role in modulating angiogenesis in the tumor environment [6, 7] and are able to secrete many proangiogenic factors such as VEGF, TNF-α, IL-8, and bFGF [7, 8, 23]. The CCN proteins are extracellular signal regulators that are well-established mediators of numerous biological processes, including angiogenesis [38]. Indeed, CCN3 has been reported to act as an angiogenic regulator in vivo [13]. Here, we showed that PCa-derived CCN3 influenced RAW264.7 macrophages and subsequently increased VEGF-dependent angiogenesis (fig. 3). These findings may improve our understanding of the relationship between increased TAM infiltration and high levels of angiogenesis in PCa, as has previously been noted [39].

CCN3 activates intracellular signaling pathways by binding to cell surface integrin receptors [25, 26, 30]. In this report, we identified components of the downstream signaling pathways involved in CCN3-induced VEGF expression. Phosphorylation of FAK, Akt, and NF-κB (IκB, IKKα/β, and p65) phosphorylation was increased in RAW264.7 cells by CCN3 treatment in a time-dependent manner (fig. 4a). In addition, pretreatment of macrophages with FAK and Akt inhibitors significantly reduced NF-κB subunit p65 translocation to the nucleus and in vivo binding of p65 to the NF-κB element on the VEGF promoter (fig. 4b and 4c). These data were supported by the results of the NF-κB reporter assay (fig. 4d and 4e). Moreover, pretreatment with the inhibitors or transfected with DN mutant of these proteins blocked CCN3-induced VEGF production by RAW264.7 macrophages and subsequent angiogenesis (fig. 4f–k). NF-κB plays multiple roles in regulating TAMs [40]. Here, we showed that NF-κB regulates CCN3-induced VEGF expression in RAW264.7 macrophages. These finding are consistent with our observation in this study and suggest that CCN3 may regulate M2 macrophage polarization through NF-κB activation. Indeed, we found that pretreatment with the pathway inhibitors abolished CCN3-induced M2 polarization of RAW264.7 macrophages (fig. S2). The molecular mechanisms underlying CCN3-induced macrophage polarization will be the subject of future investigations.

Finally, we used 2 in vivo models to determine whether PCa-secreted CCN3 could affect macrophage function and subsequently promote angiogenesis in the PCa microenvironment. In the chicken egg CAM model we found that vessel formation was increased by application of a mixture of PC3 and RAW264.7 cells compared with that induced by PC3 cells alone, but this effect was attenuated upon CCN3 knockdown in PC3 cells (fig. 5). Similarly, we found that injection of PC3 and RAW264.7 cells into SCID mice induced rapid tumor growth and angiogenesis, but this was markedly reduced by knockdown of CCN3 expression in PC3 cells (fig. 6a-d). Our results also showed that CCN3 expression was correlated with CD206+ M2 macrophage infiltration in xenograft mice model (fig. 6d). These in vivo results confirm that PCa-secreted CCN3 can regulate macrophage M2 polarization, macrophage-promoted angiogenesis in a tumor environment.

Our previous work showed that CCN3 is highly expressed in malignant PCa cells and regulates cancer cell migration [11]. Here, we show that PCa-secreted CCN3 serves as a chemoattractant and induces macrophage infiltration. However, CCN3 is a multifunctional cytokine and regulates many cell functions. We found that tumor-secreted CCN3 could skew macrophages from an M1 to an M2 phenotype, which is likely to create a more immunosuppressive tumor microenvironment and thus impair anti-tumor responses. Moreover, our data provide evidence that CCN3 increases VEGF expression and regulates angiogenesis through the FAK/Akt/NF-κB signaling pathway in macrophages. These results all support the conclusion that PCa-secreted CCN3 modulates the tumor microenvironment by regulating TAM infiltration (fig. 6e). In recent years, TAMs have attracted attention as therapeutic targets for solid tumors. Tumor-secreted factors that induce macrophage infiltration and regulate the functions of TAMs also deserve consideration. CTGF, another member of the CCN family associated with tumorigenesis, is a novel therapeutic target for the treatment of pancreatic cancer, and is currently being investigated in a Phase I clinical trial [41, 42]. Collectively, our results presented here suggest that CCN3 could be a novel therapeutic target for the treatment of PCa.

## MATERIALS AND METHODS

### Material

FAK inhibitor (FAKi), Akt inhibitor (Akti), NF-κB inhibitor (PDTC) and IκB protease inhibitor (TPCK) were purchased from Sigma-Aldrich (St. Louis, MO, USA). Recombinant human CCN3 was purchased from R&D Systems (Rocky Hill, CT, USA). All other chemicals were purchased from Sigma–Aldrich (St Louis, MO, USA). The methods of Adhesion assay, Western blot analysis, Quantitative real-time PCR, Enzyme-linked immunosorbent assay, Tube formation, Chromatin immunoprecipitation, Immunofluorescence microscopy, Luciferase reporter assay, Chick chorioallantoic membrane (CAM) assay and In vivo tumor xenograft study are provided in Supplementary Information.

### Cell culture

All cell lines were obtained from American Type Culture Collection (ATCC; Manassas, VA, USA). PCa cells were cultured in RPMI 1640 medium supplemented with 20 mM HEPES, 10% FBS, 2 mM glutamine, penicillin, and streptomycin (Invitrogen, CA, USA), and were maintained at 37°C in a 5% CO_2_ atmosphere. The murine macrophage cell line RAW264.7 was grown in Dulbecco's Modified Eagle Medium (DMEM) (Invitrogen, CA, USA) supplemented with 10% FBS, penicillin, and streptomycin. The media were changed every 48 h.

### Endothelial progenitor cells (EPCs) Cell culture

The protocol for culture of EPCs was approved by the Institutional Review Board of Mackay Medical College, New Taipei City, Taiwan (reference number P1000002), and all subjects gave informed written consent before enrollment in this study. Peripheral blood (80 ml) was collected from healthy donors, and mononuclear cells were isolated by centrifugation on Ficoll-Paque PLUS (Amersham Biosciences, Uppsala, Sweden) according to the manufacturer's instructions. CD34-positive progenitor cells were isolated from the mononuclear cell fraction using a CD34 MicroBead kit and MACS Cell Separation System (Miltenyi Biotec, Bergisch Gladbach, Germany). CD34-positive EPCs were maintained and characterized as described previously [43]. Briefly, human CD34-positive EPCs were cultured in MV2 complete medium consisting of MV2 basal medium and growth supplement (PromoCell, Heidelberg, Germany), supplemented with 20% defined FBS (HyClone, Logan, UT). The cultures were seeded on 1% gelatin-coated plasticware and maintained at 37°C in a humidified 5% CO_2_ atmosphere.

### Establishment of the CCN3 knockdown PC3 cell line

To establish the CCN3 knockdown PC3 cell line, lentiviral vectors for expression of the CCN3-specific shRNAs or a negative control (Neg) shRNA were purchased from the National RNAi Core Facility Platform (Taipei, Taiwan). The CCN3 shRNA targets sequence were CCAGAGCAGCCAACAGATAAG, CCCACCATCAAAGGAA -TATAA and CGCACCAAGAAGTCACTCAAA. Lentiviruses were prepared according to standard protocols. For infection, PC3 cells were seeded in a 6-well dish and the lentivirus was added (multiplicity of infection = 10) in medium containing polybrene (8 μg/ml). After 24 h, the culture medium was changed, and 48 h later, 1 μg/ml puromycin was added to select for Neg shRNA- and CCN3 shRNA-expressing cells. The surviving cells were selected and clonal cell populations were expanded. For monolayer growth curves, 10^4^ cells were plated in 6 well plates and grown for 1 to 6 days. Cells were trypsinized, and cell numbers was counted.

### Preparation of conditioned media

PCa cells (PC-3, DU145, LNCaP and PzHPV-7) and RAW264.7 cells (2 × 10^6^) were grown overnight in 100-mm culture dishes. The cells were washed with PBS and cultured in RPMI medium supplemented with 1% FBS for 48 h. The culture supernatants (conditioned media; CM) were collected. To normalize for differences in cell density due to proliferation, the cells from each plate were collected and the total DNA content per dish was determined (spectrophotometric absorbance, 260 nm). The RAW264.7 macrophages were incubating in PC3 Neg-CM and PC3 shCCN3 for 24h. After 24h culturing, the supernatants from RAW264.7 were collected and named as PC3 Neg-RAW CM and PC3 shCCN3-RAW CM. The CM volumes were then normalized by addition of cell culture medium to account for varying DNA content between samples.

### Immunohistochemistry (IHC)

After approval by the local ethics committee, specimens of PCa tissue and adjacent normal tissues were obtained from 21 patients with pathologically confirmed PCa who had undergone surgical resection at Taichung Veterans General Hospital. Sections (5-μm thick) of paraffin-embedded tissue were placed on glass slides, rehydrated, incubated with 3% hydrogen peroxide to quench endogenous peroxidase activity, and then blocked by incubation with 3% BSA in PBS. Sections were then incubated with the primary rabbit polyclonal anti-human CD206 antibody (Cat.#: ab64693, Abcam, MA, USA) at a dilution of 1:50 and incubated at 4°C overnight. After 3 washes with PBS, the samples were incubated with a 1:50 dilution of biotin-labeled goat anti-mouse IgG secondary antibody, and bound antibodies were detected using an ABC Kit (Vector Laboratories, Burlingame, CA, USA). The slides were stained with chromogen diaminobenzidine, washed, counterstained with Delafield's hematoxylin, dehydrated, treated with xylene, and mounted.

For CCN3, CD206, VEGF and CD31 IHC assays in the in vivo tumor xenograft model, the rabbit polyclonal antibodies against IgG (Santa Cruz Biotechnology, TX, USA), CCN3 (Cat.#: S1262, Epitomics, CA, USA), CD206 antibody (Cat.#: ab64693, Abcam, MA, USA) and VEGF antibody (Abcam, MA, USA) or anti-CD31 antibody (Epitomics, CA, USA) have been used. Tumor samples were collected from sacrificed mice and fixed in 4% paraformaldehyde in PBS for at least 72 h, dehydrated in increasing concentrations of ethanol, and embedded in paraffin. Serial sections of 5-μm thickness were cut longitudinally and incubated with anti-CCN3 antibody (1:50), anti-CD206 antibody (1:50), anti-VEGF antibody (1:50) or anti-CD31 antibody (1:100) at 4°C overnight. After 3 washes with PBS, the samples were incubated with a 1:50 dilution of biotin-labeled goat anti-mouse IgG secondary antibody, and bound antibodies were detected using an ABC Kit. The slides were stained with chromogen diaminobenzidine, washed, counterstained with Delafield's hematoxylin, dehydrated, treated with xylene, and mounted.

### Transwell migration assay

All cell migration assays were performed using Transwell inserts (8-μm pore size; Costar, NY, USA) in 24-well dishes. RAW264.7 cells were pretreated for 30 min with the indicated concentrations of inhibitors (FAKi, Akti, PDTC, or TPCK; Sigma-Aldrich, St. Louis, MO, USA) or vehicle (0.1% DMSO). Cells (1 × 10^4^ in 200 μl of serum-free medium) were then seeded in the upper chamber of the Transwell and 300 μl of the same medium containing varying concentrations of CCN3 (R&D Systems, Minneapolis, MN, USA) was placed in the lower chamber. Each experiment was performed with triplicate wells and was repeated at least 3 times.

### Statistical analysis

Data are presented as the mean ± standard error of the mean (SEM). Statistical comparisons between 2 samples were performed using the Student's t-test. Statistical comparisons of more than 2 groups were performed using one-way analysis of variance with Bonferroni's post-hoc test. A P-value of less than 0.05 was considered statistically significant.

## SUPPLEMENTARY METHODS, FIGURES, TABLES AND REFERENCES


