# Supplementary material for: Prostate cancer-derived CCN3 induces M2 macrophage infiltration and contributes to angiogenesis in prostate cancer microenvironment
Source: Oncotarget. 2014 Feb 4;5(6):1595–608. doi: 10.18632/oncotarget.1570 (PMC4039234; doi:10.18632/oncotarget.1570)
Supplement: Supplementary file 1 [file oncotarget-05-1595-s001.docx]

**Supplementary Methods**

**Adhesion assay**

The adhesion of RAW264.7 cells to PC3 cells was assayed as described previously ([1](#_ENREF_1)). Monolayers of PC3 cells (4 × 10^5^/well) in 24-well plates were treated with varying concentrations of CCN3 for 24 h. RAW264.7 cells (1 × 10^4^ cells) were labeled with 0.1 µg/ml BCECF-AM (Calbiochem, San Diego, CA, USA) and added to the PC3 monolayers. After 30 min, nonadherent cells were removed by washing with PBS and the adherent cells were immediately counted with a Leica TCS SP2 Spectral Confocal System (Leica, Wetzlar, Germany).

**Western blot analysis**

RAW264.7 cell lysates were prepared and proteins were resolved by SDS-PAGE. Proteins were transferred (400 mA, 2 h) to PVDF membranes (EMD Millipore Corporation, MA, USA), after which the membranes were blocked with 4% BSA for 1 h at room temperature (RT) and then incubated with 1:2000 dilutions of rabbit anti-human antibodies against phospho (p)-FAK, FAK, p-Akt, Akt, p-p38, p38, p-IKK, IKK, p-IκBα, IκBα, p-p65, p65, VEGF, VCAM, CCN3, or β-actin (Santa Cruz Biotechnology) for 1 h at RT. The membranes were washed 3 times for 10 min each and then incubated with a 1:2000 dilution of peroxidase-conjugated donkey anti-rabbit secondary antibody for 1 h at RT. The membranes were visualized by enhanced chemiluminescence using X-Omat LS film (Eastman Kodak, Rochester, NY, USA).

**Quantitative real-time PCR**

Total RNA was extracted from chondrocytes with a TRRzol kit (MDBio Inc., Taipei, Taiwan). The reverse transcription reaction was performed using 2

μg of total RNA (in 2

μL of RNase-free water) that was reverse transcribed into cDNA with an MMLV RT kit (Promega, Madison, WI) by following the manufacturer's instructions. The reverse transcription reaction mixture was incubated at 37°C for 60

min and then at 70°C for 5

min to inactivate MMLV. Quantitative real-time PCR (qPCR) analysis was carried out using the KAPA SYBR® FAST qPCR Kits (Kapa Biosystems, MA, USA). cDNA templates (2

μL) were added to each 25

μL reaction with the FAST qPCR mix. All primer used in qPCR were list in table s1. qPCR assays were carried out in triplicate on a StepOnePlus sequence detection system (Life technologies, CA, USA). The cycling conditions were as follows: 10

min polymerase activation at 95°C followed by 40 cycles at 95°C for 15

s and at 60°C for 60

s. The threshold was set above the nontemplate control background and within the linear phase of target gene amplification to calculate the cycle number at which the transcript was detected (denoted *C_T_*).

**Enzyme-linked immunosorbent assay**

RAW264.7 (2 × 10^4^) cells were pretreated for 30 min with varying concentrations of inhibitors (FAKi, Akti, PDTC, or TPCK) or vehicle (0.1% DMSO) and then incubated with CCN3 (0–100 ng/ml) for 24 h at 37°C. The medium was removed and stored at −80°C until VEGF levels were measured using ELISA kits (Biocompare, San Jose, CA, USA), according to the manufacturer’s instructions.

**Tube formation**

Matrigel (BD Biosciences, Bedford, MA, USA) was dissolved at 4°C, aliquots of 150 µl/well were added to 48-well plates, and the plates were incubated at 37°C for 30 min. EPCs were resuspended at 5 × 10^4^/100 µl in culture medium (50% EGM-MV2 medium and 50% CM from RAW264.7 cells incubated with 30 ng/ml CCN3), and added to the wells. VEGF (20 ng/ml) or culture medium were used as positive and negative controls, respectively. After 6 h of incubation at 37°C, EPC tube formation was assessed by microscopy and each well was photographed at ×200 magnification under a light microscope. The number of tube branches and the total tube length were calculated using MacBiophotonics Image J software.

**Chromatin immunoprecipitation**

Chromatin immunoprecipitation (ChIP) assays were performed as described ([2](#_ENREF_2)). Chromatin was prepared and incubated with an anti-p65 antibody. DNA was extracted from the immunoprecipitates, purified, and resuspended in H_2_O. Immunoprecipitated DNA was amplified by PCR using the following primers: 5′-GGGGTCACTCTAGTTGTCCCTATCCT-3′ and 5′-CCTAGGCCACTACCGCGAAATG-3′ ([3](#_ENREF_3)). PCR products were resolved by 1.5% agarose gel electrophoresis and visualized by UV light.

**Immunofluorescence microscopy**

RAW264.7 cells grown on glass coverslips were rinsed once with PBS and fixed in 3.7% paraformaldehyde for 10 min at RT. Cells were then 3 times with PBS and blocked with 4% BSA for 15 min. Cells were then incubated with rabbit anti-mouse p65 (1:100) for 1 h at RT, washed again, and incubated with FITC-conjugated goat anti-rabbit IgG for 1 h. Finally, cells were washed, mounted, and examined with a Leica TCS SP2 Spectral Confocal System.

**Luciferase reporter assay**

PC3 cells were transfected with an NF-κB reporter plasmid using Lipofectamine 2000 (Invitrogen), according to the manufacturer’s protocol. Twenty-four hours after transfection, the cells were treated with the indicated inhibitors for 30 min, and CCN3 or vehicle were then added for a further 24 h. Cell extracts were then prepared, and luciferase and β-galactosidase activities were measured.

**Chick chorioallantoic membrane (CAM) assay**

The angiogenic activity was evaluated using a CAM assay as described previously ([4](#_ENREF_4)). Briefly, fertilized chicken eggs (5 eggs/group) were incubated at 37°C in an 80% humidified atmosphere. On developmental day 8, PC3 Neg cells (2 × 10^6^), PC3 shCCN3-3 cells (2 × 10^6^), RAW 264.7 cells (2 × 10^5^), PC3 Neg cells plus RAW264.7 cells, or PC3 shCCN3-3 cells plus RAW264.7 cells were resuspended in Matrigel and placed onto CAMs for 4 days. The CAMs were then examined by microscopy and photographed. Angiogenesis was quantified by counting the number of blood vessel branches. All animal work was performed in accordance with a protocol approved by the China Medical University (Taichung, Taiwan) Institutional Animal Care and Use Committee.

***In vivo* tumor xenograft study**

Four-week-old male SCID mice were purchased from Lasco (Taipei, Taiwan) and were maintained in pathogen-free conditions. PC3 cells (1 × 10^6^) and PC3 shCCN3-3 cells (1 × 10^6^), either alone or mixed with RAW264.7 cells (1 × 10^5^) were injected subcutaneously into the flanks of SCID mice. Mice were sacrificed 28 days later and the tumors were excised, weighed, and prepared for IHC. All mice were handled in accordance with the Animal Care and Use Guidelines of the China Medical University (Taichung, Taiwan) under a protocol approved by the Institutional Animal Care and Use Committee.

**Supplementary Figure Legends**

**Figure S1. Pretreatment with CCN3 skews LPS-treated RAW264.7 macrophages from an M1 to an M2 phenotype and inhibits LPS-induced proinflammatory cytokine expression.** Innate RAW264.7 macrophages were pretreated with or without CCN3 (30 ng/ml) for 30 min and then stimulated with LPS (50 ng/ml) plus IFN-γ (20 ng/ml) for 24 h. Total RNA was extracted and CD204, CD206, Arg-1, Mrc-2, TNF-α, IL-1β, and IL-6 mRNA expression levels were examined by qPCR. Results are expressed as the mean ± SEM of triplicate samples. *P < 0.05 compared with the control treatment, # P < 0.05 compared with LPS/IFN-γ treatment.

**Figure S2. Inhibition of CCN3-induced M2 polarization by pretreatment with CCN3-elicited signal pathway inhibitors in RAW264.7 macrophages.** RAW264.7 macrophages were pretreated for 30 min with FAKi (10 µM), Akti (1 µM), PDTC (10 µM), or TPCK (1 µM) and then stimulated with CCN3 (30 ng/ml) for 24 h. Total RNA was extracted and CD204, CD206, Arg, and Mrc mRNA expression levels were examined by qPCR.


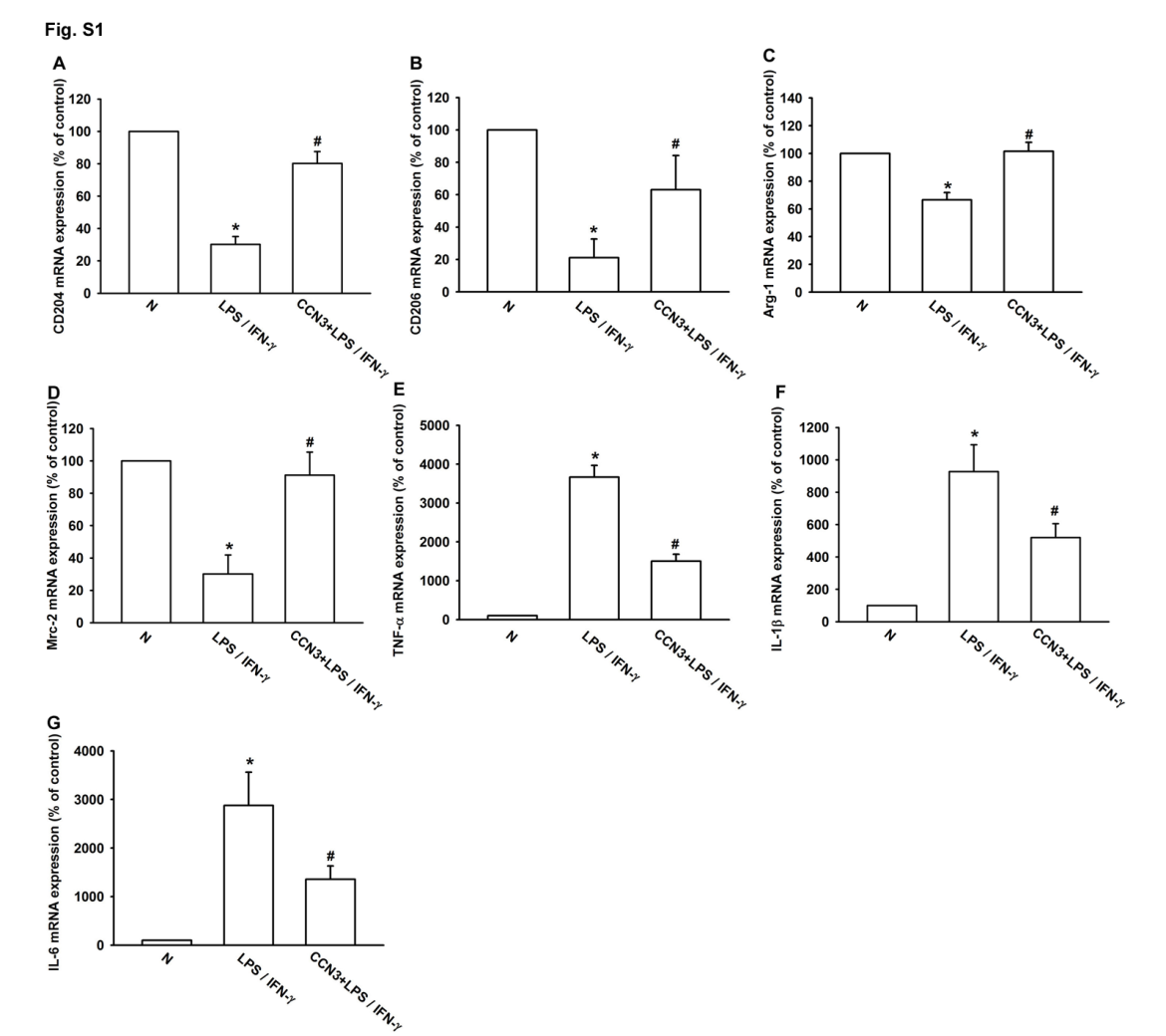





**Table. s1 Primers sequence used in qPCR assay.**

| CD204 F primer | GCACAATCTGTGATGATCGCT |
| --- | --- |
| CD204 R primer | CCCAGCATCTTCTGAATGTGAA |
| CD206 F primer | CTCTGTTCAGCTATTGGACGC |
| CD206 R primer | CGGAATTTCTGGGATTCAGCTTC |
| Arg-1 F primer | CCTATGTGTCATTTGGGTGGATG |
| Arg-1 R primer | GGTTGTCAGGGGAGTGTTGAT |
| Mrc-2 F primer | ATCCAGGGAAACTCACACGGA |
| Mrc-2 R primer | GCGCTCATCTTTGCCGTAGT |
| TNF-a F primer | CCCTCACACTCAGATCATCTTCT |
| TNF-a R primer | GCTACGACGTGGGCTACAG |
| IL-1β F primer | CTGTGACTCATGGGATGATGATG |
| IL-1β R primer | GCCTGTAGTGCAGTTGTCTAAT |
| IL-6 F primer | TAGTCCTTCCTACCCCAATTTCC |
| IL-6 R primer | TTGGTCCTTAGCCACTCCTTC |
| VEGF F primer | GCACATAGAGAGAATGAGCTTCC |
| VEGF R primer | CTCCGCTCTGAACAAGGCT |

**Supplementary Reference**

1. Kawakami A, Aikawa M, Nitta N, Yoshida M, Libby P, Sacks FM. Apolipoprotein CIII-induced THP-1 cell adhesion to endothelial cells involves pertussis toxin-sensitive G protein- and protein kinase C alpha-mediated nuclear factor-kappaB activation. Arterioscler Thromb Vasc Biol. 2007;27:219-25.

2. Chiu YC, Yang RS, Hsieh KH, Fong YC, Way TD, Lee TS, et al. Stromal cell-derived factor-1 induces matrix metalloprotease-13 expression in human chondrocytes. Molecular pharmacology. 2007;72:695-703.

3. Shima DT, Kuroki M, Deutsch U, Ng YS, Adamis AP, D'Amore PA. The mouse gene for vascular endothelial growth factor. Genomic structure, definition of the transcriptional unit, and characterization of transcriptional and post-transcriptional regulatory sequences. J Biol Chem. 1996;271:3877-83.

4. Green CE, Liu T, Montel V, Hsiao G, Lester RD, Subramaniam S, et al. Chemoattractant signaling between tumor cells and macrophages regulates cancer cell migration, metastasis and neovascularization. PloS one. 2009;4:e6713.
